# Vitamin D Deficiency Is Not Associated with Changes in Retinal Geometric Parameters in Young People with Type 1 Diabetes

**DOI:** 10.1155/2013/280691

**Published:** 2013-07-07

**Authors:** Myra Poon, Maria E. Craig, Harleen Kaur, Janine Cusumano, Muhammad Bayu Sasongko, Tien Yin Wong, Kim C. Donaghue

**Affiliations:** ^1^Institute of Endocrinology and Diabetes, The Children's Hospital at Westmead, Locked Bag 4001, Westmead, NSW 2145, Australia; ^2^Discipline of Paediatrics and Child Health, The University of Sydney, Sydney, NSW 2006, Australia; ^3^School of Women's and Children's Health, University of New South Wales, Sydney, NSW 2052, Australia; ^4^Centre for Eye Research Australia, University of Melbourne, 32 Gisborne Street, East Melbourne, VIC 3002, Australia; ^5^Department of Ophthalmology, Gadjah Mada University, Bulaksumur, Yogyakarta 5528, Indonesia; ^6^Singapore Eye Research Institute, Singapore National Eye Centre, 11 Third Hospital Avenue, Singapore 168751

## Abstract

Changes in retinal geometric parameters predict risk and progression of diabetic retinopathy (DR). We have shown that vitamin D deficiency (VDD) is associated with DR. We hypothesized that VDD mediates changes in retinal geometric parameters. Retinal vascular geometric parameters were assessed using a semiautomated computer program in photographs from young people with type 1 diabetes (T1D) (*n* = 481) and summarized as central retinal arteriolar and venular equivalents (CRAE, CRVE), fractal dimension, length-diameter ratio, branching angle and curvature tortuosity. Parameters were compared between those with and without DR and VDD (25-hydroxyvitamin D concentration ≤ 50 nmol/L). Retinal vascular geometric parameters were also compared across quartiles of vitamin D levels. Median CRVE was higher in patients with DR compared with those without (median (IQR) CRVE 247.3 **μ**m (31.3) versus 238.8 **μ**m (23.5), *P* = 0.01). Fractal dimension was marginally greater in patients without VDD (1.49 (0.06) versus 1.47 (0.07) *P* = 0.03). There was no difference in CRAE, CRVE, length-diameter ratio, branching angle, and curvature tortuosity between those with and without VDD and across quartiles of 25OHD. In conclusion, DR is associated with higher CRVE in young people with T1D; however, VDD is not associated with changes in retinal vascular geometric measures, suggesting an earlier role in the time course of DR pathogenesis.

## 1. Introduction

Despite improvements in treatment, diabetic retinopathy (DR) remains a significant complication of type 1 diabetes (T1D). Identification of early treatable predictors of DR, may allow more aggressive management of those at high risk. There is increasing evidence that vitamin D deficiency (VDD) may play a role in pathogenesis of DR. In adults with type 2 diabetes, lower 25-hydroxyvitamin D (25OHD) levels have been associated with proliferative DR [1, 2]. We have shown that VDD is associated with a twofold increased risk of DR (OR 2.12, 95% CI 1.03–4.33) independent of diabetes duration and HbA1c [3]. 

Changes in retinal microvascular geometry can be used to predict DR prior to the development of microaneurysms or hemorrhage [4, 5]. These retinal measurements include retinal arteriolar and venular calibers, vessel tortuosity, length-diameter ratio, branching angles, and fractal dimension. In the Wisconsin Epidemiology Study of Diabetic Retinopathy (WESDR), an increase in retinal venular caliber of 10 *μ*m was associated with higher 6-year incidence of DR, progression of DR, and incidence of proliferative DR [6]. Longitudinal studies from our group have demonstrated that larger retinal arteriolar caliber [7, 8] and changes in length-diameter ratio and tortuosity [9] predict the development of DR. In cross-sectional studies, increased vessel tortuosity [10] and increase fractal dimension [11] have also been associated with increased risk of DR independent of known risk factors of microvascular complications.

The mechanisms underlying these changes in retinal geometry are unclear, but may relate to endothelial cell dysfunction, neovascularization or relative tissue hypoxia [9, 10]. Calcitriol, the active form of vitamin D, has been shown to inhibit retinal neovascularization, and reduce endothelial cell viability and function in animal models [12] and adults with type 2 diabetes [13]. 

We therefore hypothesized that VDD mediates changes in retinal vascular geometry by its effects on endothelial cell function and angiogenesis and examined differences in retinal vascular caliber, fractal dimension, length-diameter ratio, branching angle, and curvature tortuosity in young people with and without DR and VDD. Since the effect of 25OHD may be nonlinear, we investigated whether there was a level at which changes in retinal vascular geometry occur by comparing patients grouped according to quartiles of 25OHD. 

## 2. Methods

We assessed 481 young people (52% male) attending the Diabetes Complications Assessment Service at the Children's Hospital at Westmead, Australia, between 2009 and 2010. Patients were defined as Caucasian/non-Caucasian according to the Australian Bureau of Statistics (ABS) standards for classifying the ethnic and cultural composition of the Australian population [14]. 

DR was assessed by a single ophthalmologist blinded to 25OHD status using digitised seven-field stereoscopic fundal photographs of both eyes and graded according to the Early Treatment Diabetic Retinopathy Study adaptation of the modified Airlie House classification. Retinal vascular geometric measures were performed using a semiautomated computer program (Singapore Vessel I Assessment, SIVA) as previously described [15, 16]. In brief, central retinal arteriolar and venular equivalents (CRAE, CRVE) were calculated from the calibers of the largest 6 arterioles and venules of the left eye, respectively. Previous studies have demonstrated high correlation between left and right eye measures [17]. Additional retinal vascular geometric measurements were also performed including length-diameter ratio (the distance from the midpoint of the first vessel branch to the midpoint of the second branch divided by the diameter of the parent vessel at the first branch), branching angle (the angle between two daughter vessels), curvature tortuosity (a measure of vessel shape and undulation), and fractal dimension (a measure of complexity of a fractal or self-similar structure). 

Total 25OHD was measured using the LIAISON analyzer (DiaSorin Inc., Stillwater, MN), and levels were adjusted for season using correction factors derived from multiple linear regression of samples taken from 550 healthy children from Sydney, Australia, as previously described [3]. VDD was defined as 25OHD <50 nmol/L [18]. Quartiles of 25OHD levels were examined to determine if there was a level at which changes in retinal vascular caliber occurred. 

## 3. Statistics

Descriptive statistics are presented as median and interquartile range (IQR). Differences between categorical variables were assessed using the chi-squared test. Differences between continuous independent variables were assessed using the Mann-Whitney *U* test. Analysis of variance (ANOVA) was used for multiple group comparisons. Analyses were performed using SPSS version 20 (IBM Corporation, Armonk, NY, USA).

## 4. Results

Total 25OHD was measured in 460/481 (96%). Median (IQR) diabetes duration was 6.7 years (4.3–9.4), and median HbA1c was 8.4% (7.6–9.4). Median 25OHD was 70.3 nmol/L (57.5–84.3), and VDD was present in 16%. There was a greater proportion of non-Caucasian patients in the group with VDD compared with those without VDD (64% versus 23%, *P* < 0.001). DR was present in 46/470 (9.8%) for whom DR grading was available.

CRAE and CRVE were measured in 454/481 (94%) of the group. Of these, 25OHD was measured in 434 patients and DR grading available in 447. Retinal vascular caliber measurements were unable to be performed in the remainder due to unavailability or unsuitability of images. Additional retinal geometric parameters were calculated in 98% of photographs (fractal dimension 471/481, complex tortuosity 472/481, branching angle, and length-diameter ration 473/481). 

Mean CRVE was greater in those with DR compared to those without (Table 1). There was no difference in mean CRAE or CRVE between those with and without VDD (Table 2). 

Total 25OHD was measured in 452/481 (94%) of those with additional retinal vascular geometric measures. Fractal dimension was marginally greater in patients who were vitamin D sufficient compared with those who were VDD (1.49 (0.06) versus 1.47 (0.07) *P* = 0.03). There was no significant difference between branching angle, length-diameter ratio, and tortuosity between those with or without VDD (Table 3) or with or without retinopathy (data not shown). 

**Figure 1:**
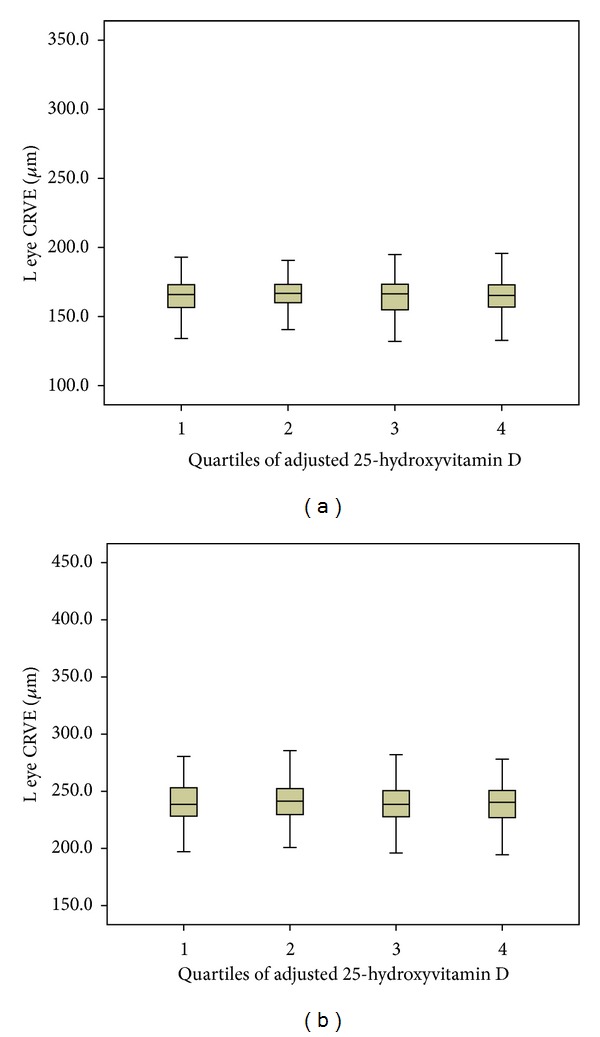
Retinal vascular calibers according to quartiles of 25-hydroxyvitamin D (25OHD). (a) Left eye central retinal arterial equivalent. (b) Left eye central retinal venular equivalent. Quartile 1: 25OHD < 57.5 nmol/L, quartile 2: 25OHD 57.6–70.3 nmol/L, quartile 3: 25OHD 70.4–84.3 nmol/L, and quartile 4: >84.3 nmol/L.

No difference in retinal vascular caliber (Figure 1), fractal dimension, branching angle, length-diameter ratio, or curvature tortuosity was seen with 25OHD quartiles (data not shown). 

## 5. Discussion

In this cross-sectional study of 481 young people with T1D, VDD was associated with a twofold increased risk of DR. However, VDD was not associated with changes in retinal vascular caliber or with differences in branching angle, length-diameter ratio, or tortuosity. In fact, normal 25OHD levels were associated with marginally greater fractal dimension. These results suggest that VDD may exert its effect at a later stage in the pathogenesis of DR or different factors may influence changes in retinal geometric parameters.

Assessment of retinal geometry has emerged as a useful biomarker of DR as well as other microvascular complications, stroke, and coronary vascular disease. Changes in retinal vascular caliber may underlie some of the pathophysiological changes associated with vascular complications [5]. We have demonstrated that mean CRVE was greater in those with DR compared with those without. In the WESDR, larger caliber venules were associated with increased 6-year incidence of DR, risk of DR progression, and incidence of proliferative DR [6]. It would appear that early increases in venular caliber persist throughout the development and progression of DR. 

These findings are in contrast to earlier longitudinal studies from our group and others demonstrating that widening of retinal arterioles is associated with an increased incidence of DR [7, 8, 19]. Our group with DR had higher CRAE (168.4 versus 165.9 *μ*m) but this was not statistically significant. The current group was older (mean age 14.9 years versus 13.5 years) and had longer diabetes duration (6.7 years versus 6.3 years) than the previous studies. It is possible that these early arteriolar changes, which may reflect early endothelial dysfunction, resolve prior to the development of features of DR. 

VDD has been implicated in the development of microvascular and macrovascular diseases [20, 21]. In animal models of retinopathy, calcitriol inhibits angiogenesis and reduces retinal endothelial cell viability, processes which are thought to be involved in the pathogenesis of DR [12]. We found that fractal dimension was marginally decreased in VDD patients, suggesting reduced angiogenesis. We did not measure parathyroid hormone (PTH) levels nor 1,25-hydroxyvitamin D levels in this cohort. It is possible that normal or elevated 1,25-hydroxyvitamin D levels, associated with elevated PTH in the VDD group, resulted in inhibition of angiogenesis. An alternative explanation is that 25OHD acts as a cofactor or promoter at an earlier time point in the development of DR, possibly via increased inflammation.

Although we did not demonstrate an association between VDD (using a level of 50 nmol/L to define deficiency) and retinal vascular caliber changes, we speculated whether there might be a level at which changes in vascular caliber would be seen. This might help to establish the level of 25OHD required to prevent the development of DR. Earlier studies have used varying levels of 25OHD to define deficiency [22–24]. However, we did not find evidence of differences in retinal geometry measures in patients grouped according to 25OHD quartiles. 

Changes in other measures of retinal vascular geometry, such as length-diameter ratio, tortuosity, and fractal dimension, are useful in the prediction of incident DR [9] and are also associated with the presence of DR [11, 15]. Identification of reversible factors associated with these early changes could be used for prevention of this significant microvascular complication. However, we did not demonstrate any association between VDD and length-diameter ratio, curvature tortuosity and branching angles. 

A possible explanation for these findings may be the decreasing incidence of DR in our clinic [25], reducing the power of the study to detect between group differences. Therefore, although the study population was relatively large, it may not have been adequate to detect a difference in retinal vascular geometric measures between those with and without VDD. Also, duration of diabetes was relatively short compared with earlier adult studies. In addition, retinal vascular caliber measurements were performed on central arterioles and venules. It is possible that vitamin D may cause changes in peripheral retinal vasculature which would not have been detected in this study. 

The strength of this study is the use of established methods in a well-characterised cohort. 

One limitation is the cross-sectional nature of this study. Further studies could examine longitudinal changes in vascular geometric parameters with presence of VDD at baseline. 

## 6. Conclusion

In young people with type 1 diabetes, retinal venular caliber was wider in those with DR compared with those without. However, although VDD was associated with a twofold increased risk in DR, VDD was not associated with a range of retinal vascular geometric measures. This suggests that VDD plays an earlier role in the pathogenesis of DR, or as a cofactor influencing changes in retinal geometric parameters.

## Figures and Tables

**Table 1:** Retinal vascular calibers in patients with and without retinopathy.

	Retinopathy (*n* = 43)	No retinopathy (*n* = 404)	*P*
Central retinal arteriolar equivalent (*µ*m)	168.4 (19.0)	165.9 (16.5)	0.74
Central retinal venular equivalent (*µ*m)	247.3 (31.3)	238.8 (23.5)	0.01

Data presented as median (IQR).

**Table 2:** Retinal vascular calibers in patients with and without vitamin D deficiency.

	Vitamin D deficient (*n* = 72)	Nonvitamin D deficient (*n* = 362)	*P*
Central retinal arteriolar equivalent (*µ*m)	165.4 (17.3)	166.3 (15.8)	1.0
Central retinal venular equivalent (*µ*m)	238.6 (27.1)	240.3 (22.8)	0.9

Data presented as median (IQR).

**Table 3:** Retinal vascular geometric parameters in patients with or without vitamin D deficiency.

	Vitamin D deficient (*n* = 73)	Non vitamin D deficient (*n* = 379)	*P*
Tortuosity (×10^−5^)	10.1 (2.5)	10.2 (2.2)	0.55
Length-diameter ratio	13.9 (5.6)	14.4 (5.7)	0.25
Branching angle (degrees)	81.4 (9.0)	81.8 (8.4)	0.39
Fractal dimension	1.47 (0.07)	1.49 (0.06)	0.03

Data presented as median (IQR).
